# Family Support in Healthy Dietary Behaviours Among Community-Dwelling Older Adults: A Scoping Review

**DOI:** 10.3390/nu18060963

**Published:** 2026-03-18

**Authors:** Pui Ying Mak, Stefanos Tyrovolas, Justina Yat Wa Liu

**Affiliations:** 1 School of Nursing, The Hong Kong Polytechnic University, Hong Kong SAR, China; pui-ying-bobo.mak@connect.polyu.hk (P.Y.M.); styrovol@gmu.edu (S.T.); 2 Department of Nutrition and Food Studies, George Mason University, Fairfax, VA 22030, USA; 3 Research, Innovation and Teaching Unit, Parc Sanitari Sant Joan de Déu, 08830 Sant Boi de Llobregat, Spain; 4 Instituto de Salud Carlos III, Centro de Investigación Biomédica en Red de Salud Mental (CIBERSAM), 28029 Madrid, Spain; 5 Research Institute for Smart Ageing, The Hong Kong Polytechnic University, Hong Kong SAR, China; 6 Research Center for Assistive Technology, The Hong Kong Polytechnic University, Hong Kong SAR, China

**Keywords:** family, family support, nutrition, diet, dietary behavior

## Abstract

**Background**: Healthy dietary behaviours are essential for maintaining health, functional independence, and quality of life in later life. Family members are a key source of social support for community-dwelling older adults, yet the ways in which family support shapes older adults’ dietary behaviours, particularly among those who retain autonomy, remain insufficiently synthesized. Therefore, this review aims to map how family support influences dietary behaviours among community-dwelling older adults by examining the forms, roles, and contextual influences of family support within a Social Support Theory framework. **Methods**: Following Joanna Briggs Institute guidance and PRISMA-ScR reporting standards, we conducted a scoping review of empirical studies published in English or Chinese. Searches were conducted across PubMed, CINAHL, PsycINFO, Web of Science, and Scopus from inception to 2025. Quantitative and qualitative evidence was synthesised using a convergent–segregated mixed-methods approach. Qualitative findings were deductively mapped to instrumental, informational, emotional, and esteem support domains. **Results**: Nineteen studies were included. Quantitative evidence indicated that family support, particularly shared meal preparation, joint dietary adherence, and autonomy-supportive encouragement, was generally associated with better diet quality, dietary adherence, and nutritional outcomes. Qualitative findings showed that the influence of family support depended on relationship dynamics and contextual factors, including communication patterns, autonomy negotiation, shared responsibility, and cultural expectations. **Conclusions**: Family support plays a multifaceted and context-dependent role in shaping dietary behaviours among community-dwelling older adults. These findings can inform the development of family-inclusive strategies and interventions that promote healthy dietary behaviours while respecting older adults’ autonomy and relational contexts.

## 1. Background

Globally, populations are ageing at an accelerating rate, resulting in a growing number of older adults living with chronic conditions and functional decline [1]. Healthy dietary behaviours, including balanced food choices, appropriate meal patterns, and quality diets, play a critical role in maintaining health, functional independence, and quality of life in later life [2,3]. Conversely, unhealthy dietary behaviours among older adults, such as inadequate protein intake and low consumption of fruits and vegetables, are associated with adverse outcomes, including malnutrition, cardiovascular disease, sarcopenia, frailty, diabetes mellitus, and increased healthcare utilization [4,5]. Maintaining healthy dietary behaviours is therefore essential for community-dwelling older adults to support healthy ageing and prevent nutrition-related morbidity [3,5].

Dietary behaviours in older adulthood are shaped by a complex interaction of individual, social, and environmental factors. Age-related physiological changes, declining appetite, chronic illness, and functional limitations may reduce dietary intake or compromise diet quality [2,6]. Age-related sensory decline, including reduced taste and smell sensitivity, may reduce appetite and food enjoyment, while oral health problems and swallowing difficulties may limit food variety and protein intake [7,8]. Polypharmacy and chronic disease management may further affect appetite, gastrointestinal comfort, and nutrient absorption [9]. Psychological factors such as depression, loneliness, and bereavement are also prevalent in later life and have been associated with irregular meal patterns and reduced dietary quality [10]. These multifaceted challenges of maintaining healthy dietary behaviours in older adulthood are closely tied to broader life transitions and contextual circumstances, making sustained healthy eating particularly complex. At the same time, social circumstances, such as living arrangements, social isolation, and inadequate social support networks, can further influence food choices, meal preparation, and eating patterns [11,12]. Among these social factors, the family represents a key source of support for many older adults living in the community [13].

Family involvement is increasingly recognized as an important determinant of health behaviours across the life course [13]. Drawing on Social Support Theory, family support can be conceptualized as encompassing emotional support (e.g., encouragement and reassurance), instrumental support (e.g., assistance with shopping, cooking, or meal preparation), informational support (e.g., advice or knowledge sharing), and appraisal support (e.g., feedback and reinforcement) [14,15]. Social Support theory was selected because it explicitly conceptualizes health behaviours as embedded within interpersonal relationships and distinguishes multiple functional types of support, making it particularly suitable for examining how family interactions shape everyday dietary practices. For community-dwelling older adults, family members may influence dietary behaviours directly through practical assistance or indirectly through shared beliefs, expectations, and social norms surrounding food and health.

The role of family support in shaping dietary behaviours among children and adolescents’ chronic conditions has been extensively examined in previous studies. For example, a systematic review of 39 randomized controlled trials found that family-based nutrition interventions for children with obesity were associated with improvements in dietary behaviours, including reduced energy intake and healthier food choices, compared with interventions without family involvement [16]. In addition, a global systematic review of 48 qualitative studies identified key family-related mechanisms influencing the eating behaviours of adolescents, such as parental modelling, family motivation, food-related rules, and parenting styles that shaped knowledge, attitudes, and practices toward healthy eating [17]. However, the relevance and manifestation of family support may differ substantially in older adulthood. Older adults typically retain greater autonomy over food-related decisions compared with children, while also facing unique age-related challenges such as declining physical capacity, widowhood, and changes in household composition [18,19,20]. Consequently, the forms of family involvement that support healthy dietary behaviours in later life may differ in nature, intensity, and acceptability from those in earlier life [12,21].

Despite growing interest in family-centred approaches to promoting health, the role of family involvement in influencing dietary behaviours among community-dwelling older adults remains underexplored. Existing reviews have largely focused on caregiver-led nutritional interventions targeting malnutrition or frailty, often emphasizing education provided to informal caregivers rather than the dynamic interaction between older adults and their family members, such as negotiation around food choices, shared decision-making, everyday communication about eating, and adaptations to changing functional or social circumstances [22,23]. Moreover, many studies prioritize clinical outcomes rather than examining how family roles, support processes, and relational contexts shape everyday dietary behaviours [6,24]. There is also limited synthesis of the barriers and facilitators influencing family involvement, such as cultural expectations, older adults’ desire for independence, family availability, and interpersonal dynamics [25,26].

Given the heterogeneity of family structures, living arrangements, and cultural contexts, a comprehensive mapping of the existing evidence is needed to clarify how families influence dietary behaviours among community-dwelling older adults. A scoping review is particularly appropriate to examine the breadth of study designs, conceptual frameworks, and methodological approaches used in this field, and to identify gaps in current knowledge [27,28].

Therefore, the aim of this scoping review is to map how family support shapes dietary behaviours among community-dwelling older adults by examining the forms, roles, and contextual influences of family support within a Social Support Theory framework. The findings from a synthesis of the existing evidence can inform the development of family-inclusive strategies to support healthy dietary behaviours and promote healthy ageing in the community.

## 2. Methods

This scoping review follows the framework of Arksey and O’Malley [29] as advanced by Levac et al. [30] and the guidance for scoping reviews from the Joanna Briggs Institute (JBI). This review was also conducted and reported in line with the Preferred Reporting Items for Systematic Reviews and Meta-Analyses Extension for Scoping Reviews (PRISMA-ScR) [28]. Our protocol is available on the Open Science Framework (https://doi.org/10.17605/OSF.IO/7EPVQ).

### 2.1. Eligibility Criteria

The inclusion and exclusion criteria were established based on the PCC (participants, concept, context) framework.

#### 2.1.1. Types of Participants

This scoping review includes studies involving community-dwelling older adults aged 60 years or above, as defined by the World Health Organization (WHO) as the threshold for an ageing population [1]. Older adults with diverse health profiles have been included, ranging from those in robust health to those with chronic conditions such as hypertension or diabetes but who retain autonomy in making daily dietary choices. Studies examining older adults and their family members, where family support is directly or indirectly related to dietary behaviours, have been included to capture the role of family support in promoting positive dietary habits. Studies focusing solely on caregivers without the direct involvement of older adults in the intervention have been excluded. No restrictions were applied to other demographic variables (e.g., gender, ethnicity, socioeconomic status) to ensure inclusivity of relevant studies.

#### 2.1.2. Concept

The core concept of this scoping review is family support in influencing healthy dietary behaviours among community-dwelling older adults. Family support is broadly defined to encompass both the active engagement of family members, including in giving emotional, informational, or instrumental support such as offering encouragement, help with meal preparation and grocery shopping, or in participating in shared meal practices; and family-related contextual factors such as living arrangements, the presence of a spouse, or the frequency of family contact, which may influence the dietary behaviours or nutritional status of older adults [10]. Studies were eligible for inclusion if they investigated how family members support or influence older adults’ dietary behaviours, food choices, or nutritional outcomes; examined relationships between family-related factors (e.g., cohabitation, family communication, or spousal support) and older adults’ diet quality or nutritional health; or explored facilitators and barriers affecting the ability of family members to support healthy eating among older adults. For this review, healthy dietary behaviours refer to eating practices that promote health and prevent disease, as characterized by adequate nutrient intake, balanced food choices, and limited consumption of harmful substances [31]. Nutritional outcomes encompass measures of diet quality, nutritional status, and related health indicators among older adults [2].

#### 2.1.3. Context

This scoping review includes studies conducted in community settings, where older adults have autonomy over their food choices, preparation, or consumption. Excluded were studies conducted in hospitals or long-term care facilities where dietary autonomy may be limited. Studies from any country were eligible, in order to provide a global perspective on family involvement in nutritional interventions for older adults. No restrictions were placed on cultural, socioeconomic, or geographic contexts to maximize the breadth of relevant findings.

#### 2.1.4. Types of Studies

Both quantitative and qualitative studies, including observation studies, quasi-experimental studies, and experimental studies, were included. Studies published in English and Chinese were included, since there might be cultural differences in dietary behaviours [27]. No limitations were placed on the year of publication.

### 2.2. Search Strategy

A three-step search strategy was employed. The search strategy was reviewed in consultation with a librarian. The first step was the initial search in PubMed and CINAHL. An analysis was conducted on the words included in the title, abstract, and keywords of the retrieved papers. Second, we searched through all of the selected databases for all words and keywords deemed to be relevant. Third, the reference lists of the selected papers were also screened for other possible related articles. The finalized search strategy is shown in Supplementary Material File S1. The search was conducted with Boolean operators (AND and OR). Free text and MeSH terms, whenever appropriate, were used. The selected electronic databases were: PubMed, CINAHL, PsycINFO, Web of Science, and Scopus. Databases were searched from inception to the year 2025.

### 2.3. Study Selection

Endnote 21 was used to manage the references, and duplications were removed. Study selection consisted of two phases. First, the two reviewers (PYM and JYWL) independently screened the titles, abstracts, and keywords of the paper to identify articles that seemed to meet the eligibility criteria. When there were disagreements over the results, the two reviewers engaged in a discussion to reach a consensus. The second phase involved retrieving and evaluating the full texts of those preliminarily selected studies in order to determine the final inclusion of articles. A flow chart was created according to the PRISMA guidelines to depict the selection process.

### 2.4. Data Extraction

Data extraction was conducted using a standardized extraction form developed by the review team to systematically capture pertinent information aligned with the aim of the review. Two independent reviewers (PYM and JYWL) extracted data from each included study, with discrepancies resolved through discussion or consultation with a third reviewer when necessary to ensure consistency and accuracy.

The extraction form captured core study details, including author(s), publication year, study design, aims and objectives, study setting, and population characteristics such as sample size and mean age, as well as family and social support variables. In addition, outcome data related to dietary behaviours and nutritional practices (e.g., food choices, meal patterns, diet quality, or dietary adherence) were collected for quantitative studies. For qualitative studies, data describing family roles, perceived facilitators and barriers to family involvement, and contextual influences on the dietary behaviours of older adults were extracted. Reported study limitations were also documented. This structured extraction process enabled a systematic comparison to be conducted across study designs and supported a subsequent quantitative summarization, qualitative thematic analysis, and integrative interpretation of the findings.

### 2.5. Critical Appraisal

The methodological quality of the included studies was assessed using the Joanna Briggs Institute (JBI) Critical Appraisal tools appropriate to each study design. Separate JBI checklists were applied for randomized controlled trials, quasi-experimental studies, cross-sectional studies, cohort studies, longitudinal studies, qualitative studies, and mixed-methods studies [32,33,34,35,36]. For the mixed-methods study, the qualitative and quantitative components were appraised independently using the corresponding JBI checklists. Two reviewers (PYM and JYWL) independently conducted the critical appraisal of all of the included studies. Any discrepancies in appraisal were resolved through discussion among the entire research team to achieve a consensus.

### 2.6. Reporting the Data

The reporting and synthesis of the data were undertaken by PYM. Data synthesis followed a convergent–segregated mixed-methods design, consistent with JBI guidance for integrating quantitative and qualitative evidence [37]. Quantitative data from observational, quasi-experimental, and randomized controlled trials, and the quantitative components of mixed-methods studies) were summarized descriptively according to study design, family support type, and outcome measures.

Qualitative data from qualitative studies and the qualitative components of mixed-methods studies were synthesized using a theory-informed deductive thematic approach guided by Social Support Theory [15]. Rather than conducting line-by-line coding, the qualitative findings were mapped against four domains of social support: instrumental, informational, emotional, and esteem support, to identify recurring patterns, facilitators, and barriers in family roles.

The quantitative and qualitative syntheses were then triangulated narratively to identify convergence and complementarity between quantitative outcomes and qualitative insights [38]. This integration provided a holistic understanding of how different forms and qualities of family support influence dietary behaviours among older adults across diverse cultural and contextual settings.

## 3. Results

### 3.1. Literature Selection Process

**Figure 1:**
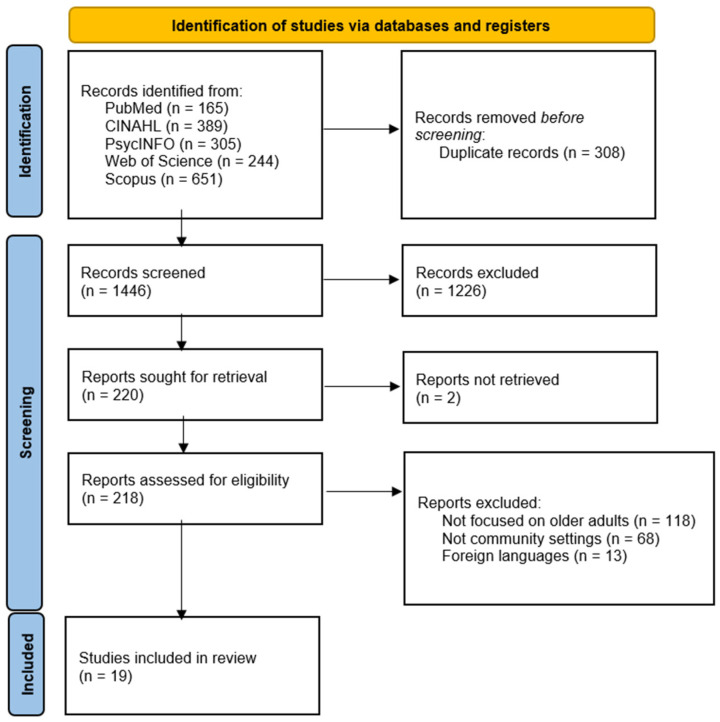
PRISMA study selection flow diagram.

A PRISMA flow diagram of each stage of the literature search is given in Figure 1. The initial search of eight databases yielded a total of 1754 publications. After the removal of duplicates, 1446 publications remained. These were screened based on their titles and abstracts, and 1226 were excluded. Of the remaining 220 articles, the full texts of 218 articles were screened, while the full texts of 2 articles could not be retrieved. Of the 218 articles, 198 did not meet the eligibility criteria for the following reasons: (a) the focus was not on older adults (*n* = 118); (b) the study did not involve a community setting (*n* = 68); (c) the study was in a language other than English (*n* = 13). In the end, 19 publications met our eligibility criteria and were included for analysis.

### 3.2. Methodological Quality

All of the included studies (*n* = 19) underwent critical appraisal using the appropriate JBI tools according to the study design (Table 1, Table 2, Table 3, Table 4, Table 5 and Table 6). Overall, the methodological quality of the evidence was acceptable, with most studies meeting the majority of the appraisal criteria.

Among the cross-sectional studies, most clearly defined the inclusion criteria, described the study settings and participants in detail, and used appropriate methods of statistical analysis [12,40,42,44,45]. However, several studies showed limitations related to exposure measurements and confounding controls, with unclear or absent strategies to address confounders reported in the studies by Ong et al. [39], Watanabe et al. [43], Chung et al. [46], and Schoenberg [47].

The single cohort study demonstrated strong methodological quality, meeting nearly all appraisal criteria, including valid exposure and outcome measurements, the identification and management of confounders, and an appropriate method of statistical analysis [48]. The longitudinal study also met most criteria, although it was unclear whether the participants were consecutively included [24].

The randomized controlled trial showed appropriate randomization and statistical analysis; however, it was unclear whether or not the participants, intervention providers, and outcome assessors were blinded, reflecting common challenges in behavioural and dietary interventions [49]. Among the quasi-experimental and pretest–posttest studies, most demonstrated a clear causal direction, consistent outcome measurements, and appropriate analyses, although one study lacked a control group [50], and the reliability of the outcome measurements was unclear in others [51,52].

The qualitative studies generally demonstrated strong congruity between the methodology, data collection, analysis, and interpretation; and the participants’ voices were well represented across the studies [47,53,54,55]. Nonetheless, reflexivity was limited, with none of the studies explicitly addressing researcher influence, and statements locating the researchers culturally or theoretically were unclear across all of the qualitative studies. Ethical approval was reported in Beverly et al. [54] and Choi et al. [55], while it was unclear whether such approval had been obtained in the studies of Gallant et al. [53] and Schoenberg [47].

### 3.3. Characteristics of the Included Studies

We identified 19 empirical studies that met our inclusion criteria and examined family roles in influencing dietary behaviours among community-dwelling older people. The evidence base comprised heterogeneous study designs, including twelve quantitative observational studies, three quasi-experimental or pretest–posttest interventions, one randomized controlled trial, three qualitative studies, and one mixed-methods study. Most studies were conducted in Asia (South Korea, Japan, Thailand, Singapore, and Indonesia) and North America (United States), with one that involved rural African American communities. Sample sizes ranged from small qualitative groups (~30–100 participants) to large observational samples (e.g., community national dataset analyses and samples comprising >300–400 participants) [39,42,46]. Populations included both healthy older adults [12,39] and older adults with chronic conditions including type 2 diabetes [24,40,43,50,53,54,55], heart failure [46], colorectal cancer [44,45], and hypertension [47,49].

Table 7, Table 8, Table 9 and Table 10 summarize the characteristics and key findings of the quantitative studies (*n* = 15), and Table 11 and Table 12 present information from the qualitative and mixed-methods studies (*n* = 4).

### 3.4. Narrative Synthesis of Quantitative Findings

Across the 15 quantitative studies that were included, diverse forms of family support were identified and mapped under the domains of the Social Support Theory: instrumental, emotional, informational, and esteem support. These studies collectively examined how family members influenced older adults’ dietary adherence, nutritional knowledge, and dietary-related behaviours in community settings or outpatient contexts. The majority of the evidence demonstrated that family support, particularly instrumental and emotional support, was positively associated with improved diet quality and better nutritional outcomes. However, the results were not consistent across the studies, as the strength and direction of these effects varied by gender, cultural norms, and the quality of support.

#### 3.4.1. Outcome Measures

Dietary and nutrition-related outcomes were measured using a wide range of self-reported and objective tools, including the Nutrition Knowledge Index [39], Diet Quality Index [44,45], Health-Promoting Lifestyle Profile—Nutrition Subscale [41], Elder Healthy Eating Scale [51], and 3-day food records [50]. Across the included studies, these tools assessed both specific targeted dietary behaviours and broader dietary practices. Specific behaviours included changes in nutrient intake and food choices, such as sodium reduction (measured by 24h urinary sodium excretion [46,49]), increased fruit and vegetable intake (reflected by serum carotenoids [48]), and protein intake (assessed via food records [50]). Broader practices encompassed adherence to healthy eating guidelines and overall diet quality (measured by diet quality indices [44,45] and the Elder Healthy Eating Scale [51]). Additional objective and biochemical measures included metabolic or clinical indicators such as HbA1c, triglycerides, BMI, cholesterol, and cardiac risk scores [40,43]. Broader health-related outcomes such as quality of life, depression, and self-esteem were also reported [41,52]. The outcome measured in the included studies captured multiple dimensions of diet-related behaviour and well-being among older adults.

#### 3.4.2. Instrumental Support: Shared Meal Preparation, Cooking, and Co-Adherence

Instrumental support, which refers to the tangible actions that family members took to help with food-related behaviours, was the most consistently reported domain. Across studies, family members engaged in meal planning, grocery shopping, cooking, and shared adherence to dietary restrictions [43,51]. Older adults who received such support generally demonstrated higher diet quality and adherence. For instance, when family members jointly followed a low-sodium diet, patients with heart failure or hypertension exhibited significantly lower 24 h urinary sodium excretion and were up to four times more likely to adhere to dietary recommendations than those without shared adherence [46,49]. Similarly, caregivers’ own healthy eating practices, such as increasing the consumption of vegetables and cutting down on foods high in sugar or fat, were associated with better diet quality and nutrient intake among colorectal cancer survivors [44]. A study conducted in rural Thailand, which involved family members in meal preparation and education sessions, led to significant improvements in the healthy eating scores of older people both one week and 12 weeks after the programme [51]. In a study conducted in Indonesia, a family-inclusive programme combining joint education and cooking sessions improved compliance to a low-salt diet and reduced levels of urinary sodium [49]. These findings suggest that the active engagement of family members in shared food-related tasks is a key facilitator of sustained dietary change.

#### 3.4.3. Emotional and Esteem Support: Encouragement, Autonomy, and Motivation

Emotional and esteem support, such as giving encouragement, showing empathy, and building confidence, emerged as another crucial influence on dietary behaviour. A study conducted by Lee et al. [40] found that older adults who perceived autonomy-supportive behaviours from their family or friends showed higher adherence to healthy diets and exercise. Similarly, older adults living with family members reported better nutrition behaviours, self-esteem, and overall health-promoting practices compared with those living alone [41].

However, the quality and tone of the emotional support that was provided determined whether its influence was positive or negative. In a study involving couples who were managing type 2 diabetes, supportive and collaborative spousal behaviours were associated with improved dietary adherence, whereas critical or controlling interactions led to lower adherence and higher levels of distress [24]. This indicates that emotional support functions effectively when autonomy and partnership are respected, but can become a barrier when it manifests as coercion or pressure.

#### 3.4.4. Informational Support: Guidance and Knowledge Sharing

Informational support, which includes providing knowledge, reminders, or practical advice, was a common but variably effective form of family involvement. In a study conducted in Singapore, older adults with access to help from family or friends showed higher scores in nutrition knowledge and were more likely to report that they understood nutritional information [39]. Two studies showed that family-based education and cooking programmes enhanced dietary knowledge among both older adults and their relatives [49,51]. However, one study indicated that passive or unsolicited advice [43] did not always translate into behavioural adherence, particularly when it was perceived as intrusive. This distinction suggests that informational support is most effective when family members are engaged as collaborative learners rather than as instructors.

#### 3.4.5. Family-Inclusive Interventions and Broader Family Context

Several quasi-experimental and intervention studies demonstrated that structured family support directly contributed to improved dietary adherence and nutrition-related outcomes among community-dwelling older adults [49,50,51,52]. In Thailand, a family-involved nutrition education programme that trained older adults and their family members in healthy meal planning, preparation, and goal setting led to significant improvements in healthy eating scores at both the short-term and 12-week follow-ups [51]. Likewise, a community-based cooking programme that encouraged spouses or caregivers to participate alongside older adults with diabetes produced reductions in total energy, fat, and sodium intake and improvements in nutrient balance [50]. A randomized controlled trial in Indonesia further supported these findings, showing that joint education and cooking sessions with family members improved compliance with a low-salt diet and reduced salt concentration in food and urine compared to standard care [49].

Living arrangements and family proximity also influenced nutritional outcomes. Older adults living with family members, particularly spouses or adult children, reported higher levels of engagement in health-promoting dietary behaviours and a lower risk of malnutrition compared with those living alone [41,42]. Frequent contact with family members was also protective against poor nutritional status, whereas isolation or limited family interaction was associated with greater vulnerability to malnutrition. These findings suggest that beyond active caregiving or shared meal practices, the presence and accessibility of family members create a supportive social environment that enables sustained healthy dietary behaviours among older adults.

### 3.5. Thematic Synthesis of Qualitative Findings

Studies with qualitative and mixed-methods findings (*n* = 4) [47,53,54,55] provided depth to the qualitative data, illuminating the mechanisms and context through which family influence operates. Family support in older adults’ dietary behaviours manifested through all four domains of the Social Support Theory. Table 13 shows a thematic synthesis of family support domains mapped to the Social Support Theory framework.

#### 3.5.1. Instrumental Support

Instrumental support encompassed practical involvement in food-related and health-related activities, including control over food preparation, shared food practices, dietary monitoring, and household assistance. Spouses, most often wives, frequently assumed responsibility for cooking and portion control, which facilitated dietary adherence but sometimes generated tension when older adults perceived a loss of autonomy or the presence of excessive control [53,54]. Collaborative practices such as joint grocery shopping, shared meal preparation, and the adoption of similar diets fostered mutual responsibility and cooperation within households [54,55].

However, instrumental support was not uniformly present. Some older adults, particularly African American participants, continued to prepare meals independently despite living with family members, reflecting limited reliance on household assistance [47]. Beyond food preparation, spouses also provided encouragement to exercise and accompaniment to medical appointments, reinforcing dietary adherence through broader health-related support [55].

#### 3.5.2. Informational Support

Informational support involved dietary competence, exchanges of health information, and health awareness. Couples actively sought dietary information from healthcare professionals, books, and the media, which enhanced their knowledge and confidence in managing dietary changes [54]. Family members with health-related backgrounds often acted as informal advisors; however, unsolicited or conflicting advice sometimes undermined adherence [53,55].

In some cases, older adults demonstrated general awareness of dietary guidelines but relied primarily on self-initiated moderation and personal responsibility, rather than on ongoing family guidance [47].

#### 3.5.3. Emotional Support

Emotional support was reflected in encouragement, empathy, emotional understanding, and emotional independence. Supportive communication, reassurance, and a sense of teamwork, particularly among couples managing chronic conditions, strengthened motivation and sustained dietary changes [54,55].

At the same time, emotional support was not always reciprocated or effective. Some participants, especially women, reported that their dietary concerns were not taken seriously by spouses, which reduced perceived support [55]. Others emphasized self-reliance and emotional independence, preferring to manage dietary behaviours without family involvement [47].

#### 3.5.4. Esteem and Appraisal Support

Esteem and appraisal support operated through patterns of communication, recognition of competence, and inclusion in decision-making. Open communication and mutual problem-solving enhanced marital relationships and dietary adherence, whereas controlling language or avoidance undermined cooperation [54,55]. Older adults valued understanding and validation, particularly from peers or family members with similar health experiences; conversely, a lack of empathy from healthy relatives contributed to feelings of isolation [53].

#### 3.5.5. Coping, Psychological Support, and Cultural Factors

Coping processes and cultural contexts further shaped family involvement in dietary behaviours. Couples often viewed chronic disease management as a shared challenge, with collaborative coping enhancing resilience and adherence [54,55]. However, overprotection, which manifested through excessive control, criticism, or nagging, sometimes undermined autonomy and self-efficacy, prompting older adults to rely instead on self-regulation and moderation strategies [47,53]. Cultural norms also influenced how support was enacted and interpreted. For example, Korean immigrant families experienced emotional and cultural strain when modifying traditional rice-based diets, whereas African American elders described culturally embedded beliefs around ageing and gradual dietary moderation [47,55].

Mapping these findings to the Social Support Theory framework reveals that while families are powerful enablers of healthy dietary behaviours, they can also inadvertently become barriers when practical or emotional support compromises autonomy or cultural identity.

These studies show that family support in dietary behaviour is complex, relational, and context-dependent rather than uniformly beneficial. Its effectiveness depends on the quality of interactions and the extent to which supportive behaviours align with the preferences, autonomy, and emotional needs of older adults. Supportive behaviours foster positive outcomes when they promote cooperation, mutual respect, and shared responsibility within families. Conversely, when family involvement becomes overly controlling, critical, or intrusive, it can undermine motivation and dietary adherence. The meaning and effect of support are also shaped by household relationships, communication patterns, and generational expectations within families.

### 3.6. Integrated Triangulation

The integrated synthesis of quantitative and qualitative evidence indicates that instrumental, emotional, and esteem support from family are most consistently associated with improved dietary behaviours in community-dwelling older adults, whereas informational support and overall family involvement show more context-dependent effects.

Quantitatively, instrumental support, such as shared meal preparation, assistance with grocery shopping, and family co-adherence to dietary recommendations, was linked to higher diet quality, better objective biomarkers (e.g., lower 24 h urinary sodium) [46,49], and improved dietary indices in family-engaged interventions [50,51]. Qualitative findings complemented the quantitative findings by showing that practical assistance facilitates healthy eating and fosters shared responsibility, although it can feel controlling when it undermines autonomy [53,54,55].

Emotional and esteem support emerged as central motivational mechanisms that facilitate sustained changes in behaviour. Quantitative studies showed that perceived autonomy-supportive behaviours from family or informal supporters were associated with higher adherence to healthy diets and greater patient activation [40,44], whereas observational studies documented poorer adherence when support took the form of pressure or criticism [24]. The qualitative findings further indicated that encouragement and respectful communication strengthened confidence and adherence, while nagging or patronizing behaviours reduced self-efficacy and sometimes led to covert non-adherence [47,53,54]. Together, these findings suggest that emotional tone and respect for autonomy shape whether family involvement supports or undermines dietary behaviours [24,43].

Informational support produced mixed outcomes across trials and observational studies. Family-inclusive education and hands-on cooking programmes increased nutrition knowledge and, in some cases, improved diet indicators [49,50,51], whereas passive advice or unsolicited tips did not reliably translate into behavioural adherence [43,47]. The qualitative findings indicate that information is more effective when shared collaboratively (e.g., learning or cooking together) rather than delivered as top-down instruction [54,55].

Contextual moderators, including living arrangement, frequency of family contact, and social participation, were supported by both quantitative and qualitative evidence. Large-sample analyses reported more active engagement in health-promoting behaviours and a lower risk of malnutrition among older adults with regular family contact or supportive co-residence arrangements [41,42], while longitudinal work linked social participation to better objective markers of diet quality [48]. The qualitative studies suggest that while proximity increases opportunities for shared meals and monitoring, it may also generate conflict when shaped by family norms, gender roles, or intergenerational tensions.

## 4. Discussion

### 4.1. Summary of the Findings

This mixed-methods scoping review mapped the nature and scope of family involvement in shaping healthy dietary behaviours among community-dwelling older adults, using a Social Support Theory framework. Across 19 empirical studies, family involvement was consistently associated with older adults’ dietary behaviours, although its effects were not uniformly positive. Instrumental and emotional/esteem support emerged as the most influential forms of family involvement, while informational support showed more variable effects. Importantly, findings drawn from both quantitative and qualitative studies converged to show that the quality of family support, particularly its respect for autonomy and collaboration, moderated whether family involvement facilitated or hindered dietary adherence. These findings suggest that family members function not merely as sources of assistance, but as key actors shaping the dietary environment, motivation, and sustainability of healthy eating in later life.

### 4.2. Interpretation of Findings and Comparison with Existing Literature

#### 4.2.1. Instrumental Support and the Role of Co-Adherence

The importance of instrumental support observed in this review is consistent with the findings from the body of research on chronic illness and ageing, which highlights the role of family members in lowering practical obstacles to healthy behaviours. Previous studies have shown that family involvement facilitates health literacy, self-care engagement, and healthier behavioural patterns among older adults, thereby shaping the broader household environment in which dietary behaviours occur [56,57]. However, this review extends prior work by differentiating between passive assistance and active co-adherence. Evidence from the quantitative studies demonstrated that when family members adopted the same dietary practices, such as following a low-sodium or balanced diet alongside the older adult—dietary adherence and objective biomarkers improved. These findings suggest that “doing with” rather than “doing for” older adults normalizes dietary change within the household and reduces environmental temptations. This is consistent with broader evidence indicating that collaborative family engagement and shared decision-making enhance adherence and satisfaction with care among older adults [58]. Earlier reviews have largely focused on education-based interventions targeting individuals [59,60]; in contrast, the present synthesis highlights that shared household practices and co-adherence may be critical for sustaining changes in dietary behaviour, particularly in the context of the long-term self-management of chronic conditions.

#### 4.2.2. Emotional and Esteem Support: Support Versus Social Control

This review has identified the boundary between supportive encouragement and counterproductive social control. While emotional involvement is frequently promoted in self-management interventions, the findings from both the quantitative and qualitative studies indicated that emotional support facilitated dietary adherence only when it respected autonomy. Studies measuring autonomy-supportive behaviours showed positive associations with diet adherence, patient activation, and reduced health risks [40], whereas another study reported poorer adherence when support took the form of pressure, criticism, or monitoring [24]. These findings resonate with previous studies suggesting that directive or coercive support may provoke behavioural reactance [61]. The qualitative findings further explained this mechanism, describing how nagging, overprotection, or patronizing communication diminished confidence and sometimes resulted in covert non-adherence [53,54]. Thus, emotional and esteem support appear to act as moderators that determine whether instrumental support is experienced as empowering or undermining.

#### 4.2.3. Informational Support and Collaborative Learning

Informational support demonstrated more diverse effects across studies. Family-inclusive education and cooking programmes improved nutrition knowledge and, in some cases, dietary indicators in the included studies [49,51]. However, passive or unsolicited advice did not reliably translate into behavioural change in another included study [43]. These findings align with prior evidence suggesting that knowledge alone is insufficient to alter entrenched dietary habits [62]. The qualitative evidence in this review clarifies this inconsistency by showing that older adults are more receptive to information when it is shared collaboratively, through joint learning, shared meal preparation, or collective goal-setting, rather than delivered in a top-down or corrective manner [55]. Informational support appears to be most useful when shared within supportive family relationships and combined with practical food-related activities, rather than provided on its own.

#### 4.2.4. Contextual Influences

The influence of family support on the dietary behaviours of older adults observed in this review is shaped by broader household and cultural contexts, and is largely consistent with research emphasizing that health behaviours are socially embedded rather than individually determined [63]. The population-based studies included in this review indicate that co-residence with family members or frequent family contact is associated with higher levels of engagement in health-promoting dietary behaviours and a lower risk of malnutrition [41,42], while greater social participation is linked to favourable objective dietary markers such as serum carotenoids [48]. These patterns can be understood in light of existing research showing that family roles and gendered divisions of food-related labour structure daily eating practices in later life, shaping both access to food and the social expectations surrounding eating [64,65,66]. In such contexts, family members often function as organizers of meals, gatekeepers of food choices, or monitors of dietary behaviour. This helps to explain why living arrangements and family proximity emerged as important correlates of dietary outcomes in this review. The findings from the qualitative studies add nuance by illustrating how contextual influences shape everyday dietary practices, rather than operating only at the level of caregiving norms. For example, shared living arrangements can increase opportunities for meal sharing and support, but they may also intensify tensions when cultural food norms or intergenerational expectations conflict with dietary recommendations [53,55]. Such tensions represent an important social challenge in multigenerational households and warrant greater attention in both research and practice [67]. The existing literature suggests that these conflicts are best managed through open communication, negotiation, and flexible meal practices that preserve older adults’ autonomy while accommodating household preferences [68]. Meal sharing does not necessarily require that all family members consume identical foods; rather, it may involve eating together while allowing variation in meal components [69]. For instance, in households where dietary preferences differ, families may prepare shared base dishes with optional additions, offer alternative protein sources, or rotate meal planning responsibilities to ensure that different preferences are respected over time. Research on autonomy-supportive family interactions indicates that collaborative decision-making and mutual respect are associated with better adherence and reduced relational strain, compared with rigid or controlling approaches [40].

Beyond these practical adaptations, a substantial body of family research demonstrates that successful navigation of dietary conflict depends on negotiation processes such as identifying and reconciling underlying interests, perspective-taking, shared problem-solving, and role flexibility within the household [70]. Studies of chronic illness management and intergenerational households have shown that families who engage in collaborative planning and explicitly discuss expectations are better able to align dietary goals with cultural traditions and personal preferences [71,72]. In contrast, unresolved tensions often arise when dietary changes are framed as unilateral prescriptions rather than shared adjustments [67]. By situating the present findings within this broader literature, it becomes clear that family-inclusive strategies must address relational dynamics directly rather than assuming that involvement alone will produce positive dietary outcomes.

Taken together, these findings suggest that the influence of family support on dietary behaviours is contingent on household dynamics and cultural context, which shape how support is enacted, interpreted, and translated into changes in dietary behaviour.

The findings of this review align conceptually with broader life-course research showing that family relationships influence dietary behaviours through relational quality, communication, and shared food practices. While most existing evidence comes from studying children and adolescents, the present review suggests that similar relational mechanisms remain relevant in later life, even when older adults retain autonomy over dietary decisions [73,74].

### 4.3. Strengths and Limitations

This scoping review has several strengths. First, it systematically mapped a diverse body of evidence from evidence drawn from quantitative and qualitative studies on family involvement in dietary behaviours among community-dwelling older adults. The inclusion of multiple study designs allowed for a comprehensive overview of how family roles, forms of support, and relational processes influence dietary behaviours across different contexts. Second, the review was guided by Social Support Theory, providing a theory-informed framework to categorize forms of family support and interpret findings in a structured and conceptually coherent manner. Third, a methodological appraisal of quality was undertaken using appropriate JBI critical appraisal tools, enabling transparent reporting of the strengths and limitations of the included evidence and supporting a cautious interpretation of the findings.

Several limitations should also be considered. As a scoping review, the primary aim was to map the breadth and nature of the existing evidence rather than to synthesize effect sizes or determine the effectiveness of interventions; therefore, conclusions regarding causality or comparative effectiveness cannot be drawn. Substantial heterogeneity across study designs, populations, outcome measures, and conceptualizations of family involvement limited direct comparison across studies and precluded the conducting of a meta-analysis. In addition, family involvement was variably defined and operationalized across studies, which may have influenced the consistency of the findings and limited the ability to draw unified conclusions on specific family roles. Finally, many of the included studies relied on self-reported dietary behaviours and family support measures, which may be subject to recall and social desirability bias. Moreover, dietary recommendations evolve over time as scientific evidence accumulates. As a result, the meaning of “healthy” dietary practices and the nature of family support aligned with these recommendations may vary across study periods and contexts. This dynamic may influence the interpretation and long-term applicability of the findings.

Despite these limitations, this review provides a comprehensive synthesis of the current evidence and highlights important gaps to inform future research and the development of family-inclusive dietary interventions for older adults in community settings.

### 4.4. Implications for Practice and Research

Globally, vertical households are becoming more prevalent, meaning that more older adults now share their living spaces with adult children or grandchildren [75]. In these shared family environments, family members play an increasingly important role in shaping everyday dietary practices [10]. The findings of this review are therefore particularly timely, as they provide insight into how family dynamics within these evolving household structures may influence healthy eating.

The findings of this review can inform the development of family-inclusive strategies to support healthy dietary behaviours and promote healthy ageing in community settings. Across the included studies, family involvement influenced dietary behaviours through practical assistance, encouragement, shared food-related practices, and communication patterns [43,45,47,54,55]. However, the review also shows that family involvement alone does not guarantee positive outcomes. Instead, the manner in which support is provided determines whether it facilitates or undermines dietary adherence. Approaches that actively engage family members while preserving the autonomy and decision-making capacity of older adults may enhance motivation, confidence, and the sustainability of dietary change.

These findings are consistent with prior interventional studies demonstrating that family-based programmes are most effective when they involve structured communication training, shared goal-setting exercises, and strategies for negotiating differences in food preferences [76,77,78]. The broader literature emphasizes that family members function not only as supporters or monitors but also as active participants in behavioural change [79,80]. Accordingly, family-inclusive strategies should include components that explicitly teach families how to manage disagreement constructively, adapt meals flexibly, and balance collective routines with individual dietary needs.

Practitioners designing family-inclusive dietary programmes should therefore consider strategies that help family members engage in ways that foster collaboration, mutual respect, and supportive communication instead of controlling or overprotective approaches. This relational perspective is particularly important in households where differing food preferences, cultural traditions, or health needs may create tensions around dietary change [81]. Supporting families to negotiate these differences constructively may help maintain both adherence and relational harmony [82].

Future research should go beyond documenting whether family support is present to examining how different family roles, interaction styles, and support processes shape dietary behaviours over time. Longitudinal and intervention studies conducted at the family or household level are needed to capture relational dynamics, negotiation of autonomy, and changes in support patterns as dietary practices evolve. By mapping how these relational processes operate specifically in the context of older adults’ dietary behaviours, the present review provides a conceptual foundation to guide future intervention design and policy development. Rather than proposing a specific programme, this synthesis highlights key relational mechanisms that should be considered when developing family-inclusive approaches to promote healthy dietary behaviours among older adults.

## 5. Conclusions

In conclusion, this scoping review demonstrates that family involvement exerts a central influence on dietary behaviours among community-dwelling older adults. Consistent with the broader literature on family and nutrition across the life course, instrumental support shapes the practical food environment, while emotional and relational support influences motivation and adherence. Family involvement is most effective when it transforms dietary management into a collaborative, respectful practice. These findings highlight the importance of designing family-engaged, autonomy-supportive strategies to promote healthy eating and well-being in later life.

## Figures and Tables

**Table 1:** Critical appraisal of the included cross-sectional studies using JBI tools.

Cross-Sectional Studies [34]								
	Were the criteria for inclusion in the sample clearly defined?	Were the study subjects and the setting described in detail?	Was the exposure measured in a valid and reliable way?	Were objective, standard criteria used for measurement of the condition?	Were confounding factors identified?	Were strategies to deal with confounding factors stated?	Were the outcomes measured in a valid and reliable way?	Was appropriate statistical analysis used?
Ong et al., 2021 [39]	Yes	Yes	Unclear	Yes	Yes	Yes	Unclear	Yes
Howell, 2019 [12]	Yes	Yes	Yes	Yes	Yes	Yes	Yes	Yes
Lee et al., 2021 [40]	Yes	Yes	Yes	Yes	Yes	Yes	Yes	Yes
Sok & Yun, 2011 [41]	Yes	Yes	Yes	Yes	No	No	Yes	Yes
Lee, Chung, & Om, 2023 [42]	Yes	Yes	Yes	Yes	Yes	Yes	Yes	Yes
Watanabe et al., 2010 [43]	Yes	Yes	Unclear	Yes	Unclear	No	Yes	Yes
Lee et al., 2019 [44]	Yes	Yes	Yes	Yes	Yes	Yes	Yes	Yes
Lee et al., 2018 [45]	Yes	Yes	Yes	Yes	Yes	Yes	Yes	Yes
Chung et al., 2015 [46]	Yes	Yes	Unclear	Yes	Yes	Unclear	Yes	Yes
Schoenberg,1997 [47]	Yes	Yes	Yes	Yes	Yes	Unclear	Yes	Yes

**Table 2:** Critical appraisal of the included cohort study using JBI tools.

Cohort Study [34]											
	Were the two groups similar and recruited from the same population?	Were the exposures measured similarly to assign people to both exposed and unexposed groups?	Was the exposure measured in a valid and reliable way?	Were confounding factors identified?	Were strategies to deal with confounding factors stated?	Were the groups/participants free of the outcome at the start of the study (or at the moment of exposure)?	Were the outcomes measured in a valid and reliable way?	Was the follow up time reported and sufficient to be long enough for outcomes to occur?	Was follow up complete, and if not, were the reasons to loss to follow up described and explored?	Were strategies to address incomplete follow up utilized?	Was appropriate statistical analysis used?
Nicklett et al., 2012 [48]	Yes	Yes	Yes	Yes	Yes	Unclear	Yes	Yes	Yes	Yes	Yes

**Table 3:** Critical appraisal of the included longitudinal study using JBI tools.

Longitudinal Study										
	Were there clear criteria for inclusion in the case series?	Was the condition measured in a standard, reliable way for all participants included in the case series?	Were valid methods used for identification of the condition for all participants included in the case series?	Did the case series have consecutive inclusion of participants?	Did the case series have complete inclusion of participants?	Was there clear reporting of the demographics of the participants in the study?	Was there clear reporting of clinical information of the participants?	Were the outcomes or follow up results of cases clearly reported?	Was there clear reporting of the presenting site(s)/clinic(s) demographic information?	Was statistical analysis appropriate?
Stephens et al., 2013 [24]	Yes	Yes	Yes	Unclear	Yes	Yes	Yes	Yes	Yes	Yes

**Table 4:** Critical appraisal of the included randomized controlled trial using JBI tools.

Randomized Controlled Trial [32]													
	Was true randomization used to assign the participants to treatment groups?	Was the allocation to the treatment groups concealed?	Were treatment groups similar at baseline?	Were participants blind to the treatment assignments?	Were those delivering treatment blind to the treatment assignments?	Were treatment groups treated identically other than during the intervention of interest?	Were outcomes assessors blind to the treatment assignments?	Were outcomes measured in the same way for all of the treatment groups?	Were outcomes measured in a reliable way?	Was follow-up complete and, if not, were differences between the groups in terms of their follow-up adequately described and analysed?	Were the participants analysed in the groups to which they were randomized?	Was an appropriate method of statistical analysis used?	Was the trial design appropriate and were any deviations from the standard RCT design (individual randomization, parallel groups) accounted for in the conduct and analysis of the trial?
Usman et al., 2023 [49]	Yes	Unclear	Yes	Unclear	Unclear	Yes	Unclear	Yes	Unclear	Yes	Yes	Yes	Yes

**Table 5:** Critical appraisal of the included quasi-experimental studies/pretest–posttest using JBI tools.

Quasi-Experimental Studies/Pretest–Posttest									
	Is it clear in the study what is the “cause” and what is the “effect” (i.e., there is no confusion about which variable comes first)?	Was there a control group?	Were participants included in any comparisons similar?	Were the participants included in any comparisons receiving similar treatment/care, other than the exposure or intervention of interest?	Were there multiple measurements of the outcome, both pre and post the intervention/exposure?	Were the outcomes of participants included in any comparisons measured in the same way?	Were outcomes measured in a reliable way?	Was follow-up complete and if not, were differences between groups in terms of their follow-up adequately described and analyzed?	Was appropriate statistical analysis used?
Archuleta et al., 2012 [50]	Yes	No	Yes	Yes	Yes	Yes	Yes	Yes	Yes
Meethien et al., 2011 [51]	Yes	Yes	Yes	Yes	Yes	Yes	Unclear	Yes	Yes
Yodmai et al., 2021 [52]	Yes	Yes	Yes	Yes	Yes	Yes	Unclear	Yes	Yes

**Table 6:** Critical appraisal of the included qualitative studies using JBI tools.

Qualitative Studies [36]										
	Is there congruity between the stated philosophical perspective and the research methodology?	Is there congruity between the research methodology and the research question or objectives?	Is there congruity between the research methodology and the methods used to collect data?	Is there congruity between the research methodology and the representation and analysis of data?	Is there congruity between the research methodology and the interpretation of results?	Is there a statement locating the researcher culturally or theoretically?	Is the influence of the researcher on the research, and vice- versa, addressed?	Are participants, and their voices, adequately represented?	Is the research ethical according to current criteria or, for recent studies, and is there evidence of ethical approval by an appropriate body?	Do the conclusions drawn in the research report flow from the analysis or interpretation of the data?
Gallant et al., 2007 [53]	Yes	Yes	Yes	Yes	Yes	Unclear	No	Yes	Unclear	Yes
Beverly et al., 2008 [54]	Yes	Yes	Yes	Yes	Yes	Unclear	No	Yes	Yes	Yes
Choi et al., 2015 [55]	Yes	Yes	Yes	Yes	Yes	Unclear	No	Yes	Yes	Yes
Schoenberg, 1997 [47]	Yes	Yes	Yes	Yes	Yes	Unclear	No	Yes	Unclear	Yes

**Table 7:** Summary of the included observational studies.

Observational Studies									
Authors & Year	Study Design	Aims	Setting	Population	Family/Social Support Variables	Outcomes	Key Findings (Family Roles)	Implications	Limitations
Ong et al., 2021 [39]	Cross-sectional study	Assessing nutrition literacy, including knowledge, competencies, and attitudes, among community-dwelling older adults in Singapore, exploring how these vary across socio-demographic factors, and identifying key predictors of better nutrition knowledge.	Eastern Singapore - outpatient clinics and community	*n* = 400; ≥65 years; mean age 71.2; 54% female; 83% Chinese; mostly married (74.2%); relatively healthy	- Leaving food decisions to others (esp. males, those with family caregivers). - Home-cooked meals more common when cared for by family. - Access to help from family/friends → higher nutrition knowledge index (NKI). - Family/friends cited as a source of nutrition information (14–20%).	Nutrition Knowledge Index (0–7). Higher scores linked to: female gender, Chinese ethnicity, ability to understand nutrition info., and access to help from family/friends.	- Males more dependent on family for food decisions. - Caregiving arrangements strongly influenced whether older adults consumed home-cooked meals and who decided on foods. - Family/friends support positively associated with better nutrition knowledge.	Nutrition interventions should involve caregivers and family, esp. for older males and minority groups. Group-based/family-inclusive strategies may improve nutrition knowledge and eating habits.	- Cross-sectional (no causality). - Healthy, educated sample (limits generalizability). - Did not assess dietary intake outcomes.
Chung et al., 2015 [46]	Multi-centre observational study (secondary analysis)	Investigating whether adherence to a low sodium diet among heart failure patients improves when family members also follow the same diet, and examining how relationship status and living arrangements influence this effect.	Outpatient cardiology clinics in Kentucky, Georgia, and Indiana (USA)	*n* = 379 outpatients with heart failure; mean age 61.7 (±11.7); 67% male; mostly Caucasian (78%); both spousal and non-spousal family contexts examined	- Family adherence to a low sodium diet (LSD) assessed via patient reports. - Relationship status (spousal vs. non-spousal). - Living arrangement (alone vs. with family).	Primary: 24 hr urinary sodium excretion (objective measure of diet adherence). Adherence defined as <3000 mg sodium/day.	- Patients whose family members also followed LSD had significantly lower sodium excretion levels and were more likely to adhere to their diet (OR = 1.6). - Adherence strongest when non-spousal family members also followed LSD (OR = 3.9). - Living with family only improved adherence if the family also followed LSD. - Simply cohabiting with family members/spouse without shared adherence did not improve outcomes.	Interventions should target patient–family dyads, encouraging family members to adopt the same diet to reinforce adherence. Family modeling and joint behaviour change are crucial in dietary interventions.	- Family adherence based on patient report (not direct measures). - Did not collect detailed data on family dynamics or reasons for non-adherence. - Observational, cannot infer causality.
Howell, 2020 [12]	Cross-sectional study	Investigating how sociocultural influences affect diet, physical activity patterns, and nutritional status among older adults living in Anchorage, Alaska.	Urban Anchorage, Alaska, USA	82 community-dwelling older adults, mean age 74, 63% female	Family influence measured using the Sociocultural Influences Survey (SIS); living with family/others	Dietary intake (FFQ, HEI), physical activity (CHAMPS, METs), anthropometrics (BMI, WtHR)	Family influence associated with increased fruit consumption and higher energy expenditure in physical activity among those living with others	Suggests family support may positively influence dietary and activity behaviours; urban programmes could leverage family involvement	Cross-sectional design, small sample, sarcopenia not assessed, limited generalizability, no intervention tested
Lee et al., 2021 [40]	Cross-sectional study	Exploring how sociocultural factors influence diet, physical activity, and nutritional outcomes among urban-dwelling older adults in Alaska.	Veterans Affairs healthcare facility (Midwestern USA)	*n* = 239 Veterans with type 2 diabetes; mean age = 61.0 yrs (SD 9.0); 97% male; majority lived with a supporter (~2/3); recruited as patients at risk for complications (elevated HbA1c and/or SBP)	Perceived autonomy support from a primary informal health supporter, measured using the Important Others Climate Questionnaire (IOCQ; α = 0.83). Supporter residence coded (in-home vs. out-of-home). Supporters = family or friends who assist ≥2×/month.	Diabetes self-care subscales (SDSCA): general diet, exercise, SMBG, foot care, medication; Patient Activation Measure (PAM); PEPPI (efficacy for provider interaction); clinical labs: HbA1c, SBP, non-HDL-C; UKPDS 5- and 10-year cardiac risk	Autonomy support significantly associated with higher adherence to general diet (*p* < 0.001) and exercise (*p* = 0.003), greater patient activation (*p* < 0.001), higher provider interaction efficacy (PEPPI) (*p* < 0.001), and lower 5 & 10 yr UKPDS cardiac risk (*p* = 0.044; *p* = 0.027). No significant association with diabetes-specific behaviours (SMBG, foot care, medication) or cross-sectional HbA1c, SBP, non-HDL-C. Interaction: autonomy support × supporter residence → patient activation (effect present among in-home supporters).	Family/friend autonomy-supportive behaviours may facilitate lifestyle behaviours (diet & exercise) and patient activation; co-residence modifies some effects (in-home autonomy support particularly important for activation). Interventions training supporters in autonomy-supportive communication (in-home and out-of-home) could be a promising strategy to improve lifestyle adherence and longer-term cardiac risk.	Cross-sectional (no causality); sample = mostly male veterans (limits broader older adult generalizability); mean age 61 but range includes younger adults (31–71)—although with a mean of ≥60, not an exclusively older sample; selection bias (participants enrolled in trial and required to name a supporter—excludes unsupported patients); outcomes partly self-reported; autonomy support is perceived (subjective) measure; multiple testing increases Type I error risk.
Nicklett et al., 2012 [48]	Secondary analysis of a longitudinal, population-based cohort	Examining how different forms of social support relate to diet quality in older women, using serum carotenoid levels as an objective indicator.	Community-dwelling older women in 12 zip codes, Baltimore, MD (USA). Baseline: 1992; follow-ups: 1993–1994	Analytic sample *n* = 325 older disabled women (≥65) who had blood drawn at follow-ups	Instrumental: satisfaction with help from family/friends (0–10); received help with preparing meals/shopping (yes/no). Emotional: having confidant; perceived need for more emotional support. Social interaction: phone call frequency; attendance at church/other activities (combined). Social space/network: frequency leaving home/neighbourhood; household composition (marital status + household size). Some measures combine family/friends. Change variables (increase/same/decrease) constructed over 1 year.	Outcome: Change in total serum carotenoids (sum of α-carotene, β-carotene, β-cryptoxanthin, lutein/zeaxanthin, lycopene) measured by HPLC. Change measured between follow-up round 1 → round 2 (1993–1994). Covariates: age, BMI change, income satisfaction.	- Baseline social support measures generally did not consistently predict carotenoid change. Help with meals/shopping did not predict improved carotenoids. - Changes in social support predicted diet change: decrease in frequency leaving home → decreased carotenoids; increase in attendance at activities → increased carotenoids (marginal). - Unexpected: decrease in satisfaction with perceived help predicted increase in carotenoids (possible coping/compensatory behaviour). - Increased frequency talking on phone predicted decrease in carotenoids. - Findings suggest social activity and family interaction (aspects of social space/interaction) are associated with maintenance of diet quality.	- Provides objective evidence linking changes in social support/social participation with diet quality—supports interventions that increase social engagement/opportunities to obtain fruit/veg (e.g., community activities, enabling mobility). - Suggests merely providing instrumental help (meal prep/shopping) may be insufficient or its effect depends on context/quality; interventions should consider autonomy, type & quality of family support (not only presence/absence). - Highlights that change (dynamic) in support matters—interventions should aim to sustain or enhance beneficial social interactions over time.	- Sample restricted to disabled, community-dwelling older women → limited generalizability to men, non-disabled older adults, other cultures/contexts. - Social support measures sometimes aggregate family & friends (hard to isolate family-specific actions). - Short follow-up period (1 year change in support and carotenoids). - Some counterintuitive associations suggest unmeasured confounding/compensatory behaviours → causality limited. - Outcome (total carotenoids) reflects fruit/veg intake only (not full diet quality). - Some sample attrition/selection (those consenting to having their blood drawn were younger).
Sok & Yun, 2011 [41]	Comparative descriptive (cross-sectional survey)	Examining and comparing physical health, self-esteem, family support, and health-promoting behaviours between older adults living alone and those living with family.	Community, Seoul, Korea	*n* = 267 community-dwelling older adults (≥65 years); 133 living alone, 134 living with family; majority female (~63%); about 57% aged 65–74, 43% ≥75	Family support scale (emotional support; 11 items, 5-point Likert, higher = better support)	Physical health status, self-esteem, health-promoting behaviours (exercise & nutrition subscales of HPLP)	- Older adults living with family reported significantly higher physical health, self-esteem, family support, and health-promoting behaviours (exercise, nutrition) than those living alone. - Family presence appears to provide emotional and motivational resources facilitating healthier lifestyles.	Findings suggest that interventions for older adults should leverage family involvement where possible, while for those living alone, alternative social support systems may be needed to promote health behaviours.	Only two HPLP subscales (exercise, nutrition) were used; convenience sample from Seoul limits generalizability; cross-sectional design prevents causal inference.
Lee et al., 2023 [42]	Cross-sectional, secondary analysis of national survey	Investigating how social detachment and related factors influence the prevalence of malnutrition among elderly residents in urban areas of South Korea.	Nationwide, South Korea, 969 districts	*n* = 10,097; ≥65 years; community-dwelling older adults; excluded those who had been institutionalized	- Living arrangements: living alone (ELA), with spouse only (ELS), with children (ELC). - Contact with children living separately (high, moderate, low). - Conjugal relationship as a form of family social capital.	Malnutrition risk measured using NSI Checklist (low, moderate, high).	- Older people living alone were most vulnerable: 16% high risk vs. 5% (spouse only) and 8% (with children). - Contact with separately living children reduced malnutrition risk, especially for ELA. - Conjugal support (spouse) had a stronger protective effect than parent–child ties.	- Highlights the protective role of spousal relationships and regular child contact in reducing malnutrition risk. - Suggests that interventions should strengthen family social capital and target high-risk groups (living alone, minimal child contact).	- Cross-sectional (no causality). - Could not specify purposes/nature of child contact. - Did not include institutionalized older people. - Limited details on how family members provide nutritional support beyond presence/contact.
Watanabe et al., 2010 [43]	Cross-sectional study	Examining the relationship between family support and glycemic control through nutritional self-care behaviours among Japanese patients with type 2 diabetes.	Kansai Electric Power Hospital, Japan	112 Japanese outpatients with type 2 diabetes; mean age 62.9 years; 61% male; avg. duration of diabetes 11.5 years	- Type of support: cooking/buying meals vs. advice/encouragement - Support provider: spouse vs. other family members - Frequency of support - Emotional response to support (appreciation vs. barriers)	HbA1c, triglycerides, BMI, cholesterol	- HbA1c lower in patients < 60 yrs with family support. - Female patients with support had lower HbA1c; male patients with support had lower triglycerides. - Active support (meal preparation) more effective than passive (advice/encouragement). - Emotional barriers linked to worse HbA1c. - Spousal support sometimes associated with poorer outcomes for older women.	- Family support can improve glycemic and metabolic outcomes, but quality, type, and perception of support matter. - Active support and positive emotional dynamics appear most beneficial, while negative or controlling support may hinder self-care.	- Cross-sectional design limits causal inference. - Single hospital, all-Japanese sample (generalizability issues). - Did not assess long-term sustainability of outcomes. - Potential unmeasured confounders (e.g., family function, socioeconomics).
Lee et al., 2019 [44]	Cross-sectional study	Examining how patient and family characteristics, perceived dietary barriers, and family efforts to improve eating habits are associated with diet quality in colorectal cancer patients.	Two National University Hospitals, South Korea	216 colorectal cancer survivors (>19 yrs; mean age 62.2; 70% male; 76% married) + their 216 primary family caregivers (mean age 55.2; 72% female; mostly spouses)	- Caregivers’ dietary strategies: modifying fat/sugar/fibre, eating more vegetables, reducing high-sugar foods - Caregiver marital status, religion - Patient-perceived barriers: knowledge gaps, expense, cravings, social eating difficulties	Diet Quality Index (DQI), daily fruit & vegetable intake, calcium intake	- Patients perceiving barriers (expense, knowledge gaps) had poorer diets. - Caregivers who themselves ate more vegetables/smaller meals and fewer high-sugar foods were linked with better patient diets (increasedF&V, increased calcium, higher DQI). - Married or religious caregivers were associated with better patient outcomes. - Women survivors were less likely to have excellent diets (gendered family dynamics).	Interventions for CRC survivors should target the patient–family dyad, addressing both patient barriers and caregivers’ behaviours. Caregiver lifestyle changes can reinforce healthier diets for survivors. Nurses and dietitians should integrate family members in dietary interventions.	Cross-sectional design (no causality). Limited to two hospitals in South Korea → generalizability issues. Focused only on CRC survivors, not the broader older adult population.
Lee et al., 2018 [45]	Cross-sectional study	Examining the association between support from family and friends for healthy eating and exercise, and improvements in self-leadership among patients with colorectal cancer.	Two National University Hospitals, South Korea	*n* = 251; CRC survivors; mean age 62.7; 61% ≥ 60 years; 69% male; 72% married	- Family encouragement of healthy eating - Family rewards, participation, criticism of exercise - Friends’ encouragement/discouragement of diet and exercise	- Self-leadership (behavioural awareness, task motivation, constructive cognition) - Exercise behaviour (≥150 min/week; maintenance ≥ 6 months) - Diet quality index (DQI)	- Family encouragement to adopt healthy eating associated with excellent diet quality and improved self-leadership. - Family rewards and participation improved both self-leadership and sustained exercise. - Friend support improved task motivation but had less impact on actual diets. - Family criticism/discouragement not effective.	- Family support is more influential than friend support for diet adoption. - Encouraging family involvement could strengthen cancer survivorship care and promote long-term changes in diet. - Programmes should leverage both family and friend support for holistic lifestyle changes.	- Cross-sectional (no causality). - Only CRC patients from 2 hospitals in Korea; generalizability limited. - Did not focus exclusively on older adults, although many participants were ≥60 years.
Stephens et al., 2013 [24]	Observational study (short-term longitudinal, 24 days)	Investigating how spouses’ daily diet-related support, persuasion, and pressure influence dietary adherence and diabetes-specific distress in older adults with type 2 diabetes, and whether these effects differ based on shared responsibility for disease management.	Community-based recruitment via clinics, media, and senior centres (USA)	*n* = 126 couples; patients with T2DM aged ≥55 (mean ~66 yrs); married/partnered; spouses without diabetes; mean years married = 38; 50% female; 76% White	- Spousal diet-related support (encouragement, appreciation, help). - Spousal persuasion (convincing, motivating). - Spousal pressure (criticism, coercion). - Shared vs. patient-only appraisal of responsibility for diabetes management.	- Daily dietary adherence (Summary of Diabetes Self-Care Activities, modified for daily use). - Daily diabetes-specific distress (Problem Areas in Diabetes scale).	- Support → increased dietary adherence from the previous day. - Persuasion → associated with decreased adherence. - Pressure → decreased adherence & increased distress. - Effects stronger in couples who appraised diabetes as a shared responsibility.	- Positive support promotes adherence, especially when illness management is viewed as shared. - Pressure/control could backfire, particularly in shared-responsibility couples. - Interventions should encourage supportive, collaborative involvement and avoid coercive strategies.	- Self-reported adherence (possible bias). - Short-term (24 days)—limited long-term insights. - Observational; causal direction uncertain. - Focus on spousal dyads only, not broader family roles.

**Table 8:** Summary of the included randomized controlled trial.

Randomized Controlled Trial										
Authors & Year	Aims	Setting	Population	Sample Size	Intervention (with Family Involvement)	Control (Without Family)	Outcomes Measured	Key Findings (Family Roles)	Implications	Limitations
Usman et al., 2023 [49]	Evaluating the impact of involving family members in educational sessions and follow-up meetings on low-salt diet compliance among hypertensive older adults.	Batua Public Health Center, Makassar, Indonesia	Older adults ≥60 yrs, diagnosed with hypertension; cognitively intact; living with a family member able to assist with self-care	*n* = 30 (15 intervention; 15 control)	Educational sessions (2 × 90–100 min, with one-week intervals) + 2 follow-up meetings (1 & 2 months after), delivered with one family member present. Content: low-salt diet education, joint cooking exercises, practical demonstrations, role of family in giving “reminders.” Based on family empowerment theory + Geragogy principles.	Usual care (monthly health check-ups provided by government), no involvement of family members.	- Compliance with low-salt diet (attitude/knowledge, subjective norms/family support, perceived obstacles) - Salt concentration in food and urine - BP (systolic, diastolic) - Family support behaviours	- Intervention group: Increasedcompliance scores (knowledge, attitudes, subjective norms), decreased perceived obstacles. - Salt concentration in food & urine significantly decreased. - Family members acted as reminders, helped with cooking modifications, reinforced adherence. - No significant change in BP, but clinical trend toward lower mean BP. - Control group: no significant improvements.	Involving family members in dietary education sessions & follow-ups improves adherence to low-salt diets among hypertensive older adults. Family acts as reminders, enforcers, and co-practitioners of diet changes. Could be scaled as community-based programmes.	Small sample (*n* = 30). Single urban setting; may not be generalizable to rural areas. BP outcomes not statistically significant. Short follow-up period (2 months).

**Table 9:** Summary of the included pretest–posttest experimental/control group studies.

Pretest–Posttest Experimental/Control Group Studies										
Authors & Year	Study Design	Aims	Setting	Population	Sample Size	Intervention/Focus	Family Role/Component	Outcomes Measured	Key Findings	Limitations
Meethien et al., 2011 [51]	Pretest–posttest experimental and control group design	Evaluating the effectiveness of a nurse-led nutritional education programme involving both older adults and their family members, in promoting healthy eating among older adults in rural northeastern Thailand.	Two rural villages, Northeastern Thailand	Older people ≥60 yrs (mean ~67), living with family; predominantly low-income farmers; Buddhist	166 elders + 166 family members (EG: 43 elders + 43 family; CG: 40 elders + 40 family)	3-month nurse-led nutritional education programme based on Pender’s HPM: group teaching, individual counselling, motivation, family support training, printed handouts, home visits.	Family members actively engaged in meal planning, preparation, overcoming barriers, goal-setting, monitoring; received training on roles and responsibilities to support older people.	Elder Healthy Eating Scale (food selection, preparation, consumption), repeated measures at baseline, 1 week, and 12 weeks post-programme.	EG had significantly higher scores in overall healthy eating and all sub-dimensions vs. CG, both at 1 week and 12 weeks post-programme; family support and self-efficacy were key success factors.	Limited to 2 rural villages, mostly farmers; supportive families only; findings may not be generalizable to older people without family support or in different socioeconomic contexts.
Archuleta et al., 2012 [50]	Quasi-experimental, pretest–posttest	Assessing whether cooking classes incorporating nutrition education and hands-on meal preparation improve nutrient intake patterns in individuals with type 2 diabetes.	Community locations in New Mexico (schools, churches, senior centres)	Adults with type 2 diabetes (mean age 63; range 30–85; 78% female; 34% Hispanic; diverse SES)	*n* = 117 (20 in 2002; 97 in 2006–2007)	Kitchen Creations (4-session diabetes cooking school, 3 hrs each). Culturally tailored nutrition education + hands-on cooking + shared meals. Based on Social Cognitive Theory.	Family members (spouses/caregivers) invited to attend and cook together, reinforcing social support and shared responsibility in dietary change.	3-day food records (energy, macronutrients, saturated fat, cholesterol, sodium, carbohydrate, fiber, sugar); pre/post comparisons.	Significant decreases in energy intake, fat, saturated fat, cholesterol, sodium, and carbohydrates (*p* < 0.05). Increase in % of calories from protein. Improvements most notable among low-income participants for cholesterol, and high-income participants for sodium.	- Quasi-experimental, no control group. - Reliance on self-reported 3-day food records. - Follow-up limited to 1 month. - Did not directly measure social support or metabolic outcomes. - Results may not be generalizable beyond New Mexico.

**Table 10:** Summary of the included quasi-experimental study.

Quasi-Experimental Study									
Authors & Year	Aims	Setting	Population	Sample Size	Intervention/Focus	Family Role/Component	Outcomes Measured	Key Findings	Limitations
Yodmai et al., 2021 [52]	Assessing the effects of a family-involved ageing network health promotion programme on reducing depression and improving quality of life among older adults in rural Thailand.	Rural communities, Khon Kaen Province, Thailand	Community-dwelling older adults aged 60–80 with chronic diseases (HTN, diabetes, hyperlipidemia, heart disease), living with family members aged 20–59.	*n* = 110 (55 intervention, 55 comparison)	Older Family Network Programme (12 months): family-inclusive health promotion programme based on the Theory of Planned Behaviour & the concept of social networks. Included training on healthy food, exercise, emotional management, disease/disability prevention. Family members joined monthly sessions.	- Family members trained as supporters and caregivers. - Family role in food preparation, emotional support, and health care at home. - Perceptions of care from family, family support, and family relationship were measured.	- WHOQOL-OLD (quality of life). - 30-item GDS (depression). - Family support, perception of health care from family, family relationship.	- At 12 months, intervention group had significantly higher QOL (overall, social participation, intimacy, physical function) and reduced depression compared to controls. - Social support and perception of family care improved significantly at 9 and 12 months. - Demonstrated that structured family involvement improved long-term wellbeing in older adults.	- Limited to rural older adults; may not be generalizable to urban populations. - Self-reported outcomes (possible bias). - Convenience sampling. - Did not assess cost-effectiveness. - Excluded frail or cognitively impaired adults.

**Table 11:** Summary of the included qualitative studies.

Qualitative Study							
Authors & Year	Aims	Setting	Population	Family Support	Key Findings (Family Roles)	Implications	Limitations
Gallant et al., 2007 [53]	Exploring how an individual’s social environment can either support or hinder the self-management of chronic illnesses.	Upstate New York, USA (community settings)	*n* = 84; ≥65 years; African American and White older adults with arthritis, diabetes, and/or heart disease. Groups stratified by race and gender. (13 focus groups)	Family roles: meal preparation, medication reminders, transport, accompanying to doctors, shared diets, encouragement, overprotection, unwanted advice, conflicting food needs. Friends: companionship for walking, shared information, emotional support, transport.	- Family = main source of tangible help but also major source of negative influences (tempting foods, discouragement, overprotection). - Friends = emotional support, shared activity, exchanges of practical information. - Positive influences more frequent than negative overall. - Gender differences: men relied more on wives; women more on children/friends.- Illustrates dual role of family: both facilitator and barrier.	Interventions should acknowledge family as both a support and hindrance; include strategies to manage negative influences. Friends are underutilized but may be effective peer support resources.	Limited generalizability (only African American and White participants, small subgroup sizes). Qualitative design—cannot measure effect sizes.
Beverly et al., 2008 [54]	Exploring how aspects of spousal relationships influence the link between self-efficacy and dietary adherence in adults with diabetes, highlighting the role of partners in supporting or hindering changes in health behaviour.	Hershey Medical Center & affiliated clinics, Pennsylvania, USA	30 married/cohabiting couples (≥50 years) mean age 65.4, where one partner had been diagnosed with type 2 diabetes within the past year (6 focus groups with couples)	Spousal involvement: control over food (shopping, preparation, monitoring), dietary competence (knowledge, skills, willingness), commitment to provide support, spousal communication, coping strategies within the marriage.	Spouses played a dual role: (1) positive—providing tangible and emotional support, dietary knowledge, reinforcement, shared responsibility; (2) negative—excessive control, nagging, conflict, overstepping autonomy. Communication quality shaped whether spousal involvement was supportive or undermining.	Highlights the importance of designing nutrition interventions that involve spouses as partners in dietary management. Interventions should recognize both the benefits and potential tensions in spousal support dynamics.	Focused only on couples with a recent diabetes diagnosis; not specific to sarcopenic obesity. Limited transferability beyond spousal dyads (excludes other family roles). Small, regional U.S. sample.
Choi et al., 2015 [55]	Exploring key domains of spousal support for diabetes self-management among Korean seniors and their spouses, using focus groups to identify support strategies and gender-related differences.	Korean community in Los Angeles, USA	*n* = 33; Korean immigrants ≥60 years with type 2 diabetes (mean age of patients 68; spouses 74); all Korean-speaking; recruited from clinics & a health information centre (5 groups: 2 with patients, 3 with spouses)	Domains of spousal support: diet management, exercise, emotional support, medical regimen, communication with HCPs, providing information; examined gender and patient vs. spouse perspectives.	- Diet was the most frequently discussed domain and major source of conflict (esp. rice consumption). - Spouses emphasized emotional support more, but patients did not acknowledge it. - Gender differences: women patients received less informational or treatment support; men less emotional support. - Support often described as “nagging” and not listened to. - Both patients and spouses stressed the importance of individualizing support and viewing diabetes care as teamwork.	- Highlights cultural and gendered dimensions of spousal roles in dietary management. - Interventions should tailor spousal support training, emphasizing teamwork, individualization, and culturally sensitive dietary strategies.	- Small, non-random sample; limited to first-generation Korean immigrants in one community. - Findings may not be generalizable to all Korean seniors or other ethnic groups.

**Table 12:** Summary of the included mixed-methods study.

Mixed-Methods Study									
Authors & Year (Title)	Study Design	Aims	Setting	Population	Family/Social Support Variables	Outcomes	Key Findings (Re: Family Roles)	Implications	Limitations
Schoenberg, 1998 [47]	Mixed-methods (survey + in-depth interviews)	Exploring the relationship between perceived social support and dietary adherence among rural-dwelling African American seniors with hypertension, aiming to understand why strong social support may not always translate into dietary compliance.	Rural community, southeastern USA	*n* = 41 African American older people with hypertension; mean age ≈ 70 s (range ~60–92)	Measured social support (Norbeck Social Support Questionnaire, IPRI); Sources included daughters (71%), sisters (63%), sons (56%), spouses (44%), grandchildren (39%); examined household composition, assistance with shopping/cooking, meal sharing	Adherence to 3 anti-hypertensive diet recommendations: fat, sodium, and weight control	Family roles included: (1) Grandchildren motivating dietary change (“desire to see grandchildren grow up”), but also undermining it by offering unhealthy foods; (2) Sisters cooking for frail siblings; (3) Spouses sometimes listed as not supportive or even obstructive; (4) Many elders reported doing shopping/cooking alone → low reliance on family.	Family involvement can be both supportive (motivation, caregiving) and obstructive (temptations, undermining behaviours). Family influence is highly contextual, not uniformly positive. Highlights cultural and social dynamics in older African-Americans.	Small sample size (*n* = 41), all African American, rural setting → limits generalizability; high baseline perception of support reduced variability; standardized support measures may not capture culturally specific or “nontraditional” supports (e.g., deceased relatives, religious faith).

**Table 13:** Thematic synthesis of family support domains.

Social Support Domain	Theme	Summary of Findings	Study Source
Instrumental Support	Control over Food	Spouses (mostly wives) controlled food preparation and portion sizes; men with diabetes felt loss of autonomy and frustration.	Beverly et al. (2008) [54]
	Shared Food Practices	Couples or families collaborating in grocery shopping, cooking, and shared diets promoted adherence and mutual responsibility.	Beverly et al. (2008) [54]; Gallant et al. (2007) [53]; Choi et al. (2015) [55]
	Dietary Monitoring and Meal Preparation	Family members (often wives or daughters) cooked healthy meals and monitored adherence, although excessive control or differing diets caused tension.	Gallant et al. (2007) [53]; Choi et al. (2015) [55]
	Household Assistance and Food Procurement	Most African American older adults reported preparing meals independently despite living with family, indicating limited instrumental support in dietary adherence.	Schoenberg (1998) [47]
	Exercise and Medical Support	Spouses encouraged or joined in exercise and accompanied patients to visits to the doctor, enhancing adherence.	Choi et al. (2015) [55]
Informational Support	Dietary Competence	Couples sought information from books, media, and healthcare professionals, improving diet knowledge and confidence.	Beverly et al. (2008) [54]
	Exchanges of Health Information	Family or friends shared illness-related information; family members with medical backgrounds acted as advisors, but unsolicited advice was sometimes unhelpful.	Gallant et al. (2007) [53]; Choi et al. (2015) [55]
	Health Awareness and Personal Responsibility	Participants expressed general awareness of dietary guidelines (e.g., fat, sodium), but often relied on self-initiated moderation rather than family advice.	Schoenberg (1998) [47]
Emotional Support	Commitment and Encouragement	Emotional reassurance, empathy, and teamwork between couples motivated adherence.	Beverly et al. (2008) [54]; Choi et al. (2015) [55]
	Emotional Understanding	Spouses provided comfort and empathy; however, women often reported their advice was “not listened to”, particularly regarding diet and exercise.	Choi et al. (2015) [55]
	Emotional Independence	Many older African American participants emphasized self-reliance and a “do-it-myself” attitude toward dietary management, reflecting emotional independence rather than reliance on family.	Schoenberg (1998) [47]
Esteem/Appraisal Support	Spousal Communication	Open communication and mutual problem-solving improved marital quality and dietary adherence; controlling talk or avoidance hindered cooperation.	Beverly et al. (2008) [54]; Choi et al. (2015) [55]
	Understanding vs. Lack of Understanding	Understanding from peers or similarly ill friends enhanced adherence, while lack of empathy from healthy family members fostered isolation.	Gallant et al. (2007) [53]
Coping/Psychological Support	Coping with Chronic Disease	Couples viewed chronic disease as a shared challenge; teamwork enhanced resilience, while lack of support led to stress and isolation.	Beverly et al. (2008) [54]; Choi et al. (2015) [55]
	Overprotection and Independence	Overprotection from adult children or spouses both supported and hindered autonomy; excessive control reduced self-efficacy.	Gallant et al. (2007) [53]; Choi et al. (2015) [55]
	Moderation and Self-Regulation	Participants managed their diet by self-imposed moderation rather than family enforcement, suggesting self-driven coping strategies.	Schoenberg (1998) [47]
Cultural Factors	Cultural Adaptation of Diet	For Korean immigrants, modifying traditional rice-based diets caused emotional and cultural strain within families; teamwork and individualized support improved adaptation.	Choi et al.(2015) [55]
	Cultural Food Beliefs and Ageing	African American elders described reducing “greasy” or “rich” foods naturally with age, reflecting culturally embedded health wisdom.	Schoenberg (1998) [47]

## Data Availability

No new data were created or analysed in this study. Data sharing is not applicable to this article.

## Supplementary Materials

The following supporting information can be downloaded at: https://www.mdpi.com/article/10.3390/nu18060963/s1, File S1: Search strategy.
