# Supplementary material for: Family Support in Healthy Dietary Behaviours Among Community-Dwelling Older Adults: A Scoping Review
Source: Nutrients. 2026 Mar 18;18(6):963. doi: 10.3390/nu18060963 (PMC13029354; doi:10.3390/nu18060963)
Supplement: Supplementary file 1 [file nutrients-18-00963-s001.zip › nutrients-4170497-supplementary.pdf]

Search strategy

PubMed

Search Set	Search Terms
#1	("Aged"[MeSH] OR "Aged, 80 and over"[MeSH]) OR ("older adult*"[tiab] OR "older people"[tiab] OR "elder*"[tiab] OR "senior*"[tiab] OR "geriatr*"[tiab])
#2	("Diet"[MeSH] OR "Dietary Supplements"[MeSH] OR "Nutrition Therapy"[MeSH] OR "Feeding Behavior"[MeSH]) OR ("nutri*"[tiab] OR "diet*"[tiab] OR "eating behav*"[tiab] OR "eating habit*"[tiab] OR "food habit*"[tiab])
#3	("Family"[MeSH] OR "Caregivers"[MeSH])
#4	("Social Support"[MeSH] OR "Family"[tiab] OR "familie*"[tiab] OR "spouse*"[tiab] OR "child*"[tiab] OR "sibling*"[tiab] OR "caregiver*"[tiab])
#5	("meal preparation"[tiab] OR "nutrition education"[tiab] OR "encourag*"[tiab] OR "emotional support"[tiab] OR "informational support"[tiab] OR "tangible support"[tiab] OR "instrumental support"[tiab] OR "nutritional intervention*"[tiab] OR "nutritional care"[tiab] OR "family nutrition therapy"[tiab] OR "Dietetic recommendations"[tiab])
#6	#1 AND #2 AND #3 AND #4 AND #5

CINAHL

Search Set	Search Terms
#1	(MH "Aged+") OR (MH "Elderly+") OR (MH "Aged, 80 and Over") OR TI ("older adult*" OR "older people" OR elder* OR senior* OR geriatric*) OR AB ("older adult*" OR "older people" OR elder* OR senior* OR geriatric*)
#2	(MH "Diet+") OR (MH "Diet Therapy+") OR (MH "Nutrition+") OR (MH "Nutrition Therapy+") OR (MH "Feeding Behavior") OR TI (nutri* OR diet* OR "eating behav*" OR "eating habit*" OR "food habit*") OR AB (nutri* OR diet* OR "eating behav*" OR "eating habit*" OR "food habit*")
#3	(MH "Family") OR (MH "Caregivers")
#4	(MH "Social Support") OR TI ("social support" OR family OR familie* OR spouse* OR child* OR sibling* OR caregiver*) OR AB ("social support" OR family OR familie* OR spouse* OR child* OR sibling* OR caregiver*)
#5	TI ("meal preparation" OR "nutrition education" OR encourag* OR "emotional support" OR "informational support" OR "tangible support" OR "instrumental support" OR "nutritional intervention*" OR "nutritional care" OR "family nutrition therapy" OR "dietetic

	recommendation*") OR AB ("meal preparation" OR "nutrition education" OR encourag* OR "emotional support" OR "informational support" OR "tangible support" OR "instrumental support" OR "nutritional intervention*" OR "nutritional care" OR "family nutrition therapy" OR "dietetic recommendation*")
#6	#1 AND #2 AND #3 AND #4 AND #5

PsycINFO

Search Set	Search Terms
#1	(DE "Family" OR DE "Caregivers")
#2	(DE "Nutrition" OR DE "Dietetics" OR DE "Eating Behavior" OR DE "Eating Habits" OR DE "Feeding Behavior") OR TI (nutri* OR diet* OR "eating behav*" OR "eating habit*" OR "food habit*") OR AB (nutri* OR diet* OR "eating behav*" OR "eating habit*" OR "food habit*")
#3	(DE "Aged" OR DE "Older Adults" OR DE "Geriatrics" OR DE "Elder Care") OR TI ("older adult*" OR "older people" OR elder* OR senior* OR geriatric*) OR AB ("older adult*" OR "older people" OR elder* OR senior* OR geriatric*)
#4	#1 AND #2 AND #3

Web of Science

Search Set	Search Terms
#1	Topic: "older adult*" OR "older people" OR elder* OR senior* OR geriatric*
#2	Topic: nutri* OR diet* OR "eating behav*" OR "eating habit*" OR "food habit*" OR "dietary pattern*"
#3	Topic: family OR familie* OR spouse* OR spousal OR partner* OR child* OR offspring OR sibling* OR caregiver* OR carer*
#4	Topic: "social support" OR "family support" OR "spousal support" OR "emotional support" OR "informational support" OR "tangible support" OR "instrumental support"
#5	#1 AND #2 AND #3 AND #4

Scopus

Search Set	Search Terms
#1	TITLE-ABS-KEY ("older adult*" OR "older people" OR elder* OR senior* OR geriatric*)
#2	TITLE-ABS-KEY (nutri* OR diet* OR "eating behav*" OR "eating habit*" OR "food habit*")
#3	TITLE-ABS-KEY (family OR familie* OR spouse* OR child* OR sibling* OR caregiver*)

#4	TITLE-ABS-KEY ("social support" OR family OR familie* OR spouse* OR child* OR sibling* OR caregiver*)
#5	TITLE-ABS-KEY ("meal preparation" OR "nutrition education" OR encourag* OR "emotional support" OR "informational support" OR "tangible support" OR "instrumental support" OR "nutritional intervention*" OR "nutritional care" OR "family nutrition therapy" OR "dietetic recommendation*"
#6	#1 AND #2 AND #3 AND #4 AND #5
